# Nectar Analysis Throughout the Genus *Nicotiana* Suggests Conserved Mechanisms of Nectar Production and Biochemical Action

**DOI:** 10.3389/fpls.2018.01100

**Published:** 2018-07-30

**Authors:** Fredy A. Silva, Adel Guirgis, Robert Thornburg

**Affiliations:** ^1^Department of Biochemistry, Biophysics and Molecular Biology, Iowa State University, Ames, IA, United States; ^2^Department of Molecular Biology, Genetic Engineering and Biotechnology Research Institute, University of Sadat City, Sadat City, Egypt

**Keywords:** *Nicotiana*, floral nectar, nectaries, carbohydrate in nectar, nectary carotenoids, hydrogen peroxide, proteins in nectar

## Abstract

We have evaluated the floral nectars of nine species from different sections of the genus *Nicotiana*. These nine species effectively cover the genus. We found that the nectary glands from these different species showed similar developmental regulation with swelling of nectaries during the first half of development and a distinct color change in the nectary gland as development approaches anthesis. When we examined the composition of the nectar from these nine different species we found that they were similar in content. Carbohydrate compositions of these various nectars varied between these species with *N. bonariensis* showing the highest and *N. sylvestris* lowest level of sugars. Based upon the amount of carbohydrates, the nectars fell into two groups. We found that hydrogen peroxide accumulated in the nectars of each of these species. While all species showed the presence of hydrogen peroxide in nectar, the quantitative amounts of hydrogen peroxide which was very high in *N. rustica* and *N. bonariensis*, suggesting be a common characteristic in short flower *Nicotiana* species. We further found that the antioxidant ascorbate accumulated in nectar and β-carotene accumulated in nectaries. β-carotene was most high in nectaries of *N. bonariensis*. We also examined the presence of proteins in the nectars of these species. The protein profile and quantities varied significantly between species, although all species have showed the presence of proteins in their nectars. We performed a limited proteomic analysis of several proteins from these nectars and determined that each of the five abundant proteins examined were identified as Nectarin 1, Nectarin 3, or Nectarin 5. Thus, based upon the results found in numerous species across the genus *Nicotiana*, we conclude that the mechanisms identified are similar to those mechanisms found in previous studies on ornamental tobacco nectars. Further, these similarities are remarkably conserved, throughout the genus *Nicotiana*.

## Introduction

The floral nectary is a unique organ that undergoes a complex developmental pathway. Over the past two decades, we have investigated the biochemistry of floral nectar and the floral nectary gland. These studies have focused on an interspecific cross of *Nicotiana langsdorffii* × *N. sanderae* (LxS8). This cross has a number of advantages that permit the biochemical analysis of these tissues. First, plants of this cross have very large nectary glands and produce copious quantities of floral nectar. This has permitted large-scale biochemical analyses of both floral nectar and the floral nectary gland. These studies have shown the floral nectar contains a limited array of proteins termed Nectarins (Carter et al., 1999; Carter and Thornburg, 2004a; Naqvi et al., 2005; Park and Thornburg, 2009). These nectar proteins function together in a novel biochemistry pathway termed the Nectar Redox Cycle. The nectar redox cycle is an oxidative cycle that produces very high levels of hydrogen peroxide as a defense compound (Carter and Thornburg, 2004a).

In nectaries of *Nicotiana*, this oxidative process is initiated by a NADPH oxidase (Carter et al., 2007) that produces high levels of superoxide (Thornburg et al., 2003) and subsequently, the Nectarin 1 superoxide dismutase in Nectar Redox Cycle pathway converts the superoxide into high levels of hydrogen peroxide, up to 4 mM, in nectar (Carter and Thornburg, 2000), that is toxic to multiple microorganisms (Carter et al., 2007). These levels of hydrogen peroxide serve to protect flowers from microbial infections (Thornburg et al., 2003).

In addition, we have also characterized the biochemistry of the nectary gland during floral development. These studies have shown that the nectary glands accumulate very high levels of photosynthate that is stored as starch during the first 4–5 days of floral development which is termed the growth phase (Ren et al., 2007a). Subsequently, about floral stage 9, (about 24 h before anthesis) there is shift in metabolism from starch anabolism to starch catabolism (Ren et al., 2007a) that results in the release of high levels of free sugar that flows into the biosynthesis of antioxidants (ascorbate and β-carotene) (Ren et al., 2007b) and into nectar via the sugar transporter SWEET9 (Lin et al., 2014). This shift in metabolism is transcriptionally controlled by a novel floral transcription factor, MYB305 (Liu et al., 2009). MYB305 is expressed about floral stage 6 (Liu et al., 2009), prior to the metabolic switch that leads to starch breakdown and sugar production. Of note, knockdown of the MYB305 protein in floral nectaries results in plants with reduced production of antioxidants as well as reduced levels of sugar in floral nectar.

Accompanying this maturation process, the nectary morphs into the plant’s premier secretory organ. The primary component of nectar secretions is a carbohydrate-rich material as a reward for pollinator activity. In *Nicotiana* plants the secretion of nectar begins about floral stage S10, and reaches a maximum at floral stage S12. There are three main carbohydrates that make up the nectar of most species (Bolten et al., 1979). The carbohydrates produced not only enter the secretion pathway to form nectar, but carbohydrates such as glucose, can also act as precursors for the biosynthesis of important nectar/nectary compounds such as ascorbate, oxalate and β*-carotene* that are crucial in redox metabolism (Horner et al., 2007).

In addition to compensating pollinators for visiting, tobacco nectar also shows defensive properties (Thornburg et al., 2003) while some of these defenses are related to redox activity (Carter and Thornburg, 2004b,c). There are also proteinaceous defenses in the genus *Nicotiana* (Carter and Thornburg, 2000, Carter and Thornburg, 2004a). The major nectar protein (*NEC1*) begins to be expressed about floral Stage 10 (Carter et al., 1999) and nectar secretion itself begins prior to Stage 11 (Ren et al., 2007b). Nectar from the interspecific cross produces a limited array of proteins that function together to a developmental NADPH oxidase is expressed initiating the Nectar Redox Cycle (Carter and Thornburg, 2004a) just before anthesis at Stage 12 (Carter et al., 2007).

To extend these observations, we have also examined the nectar biochemistry from *Petunia* sp., a close relative of tobacco. Those studies demonstrated that the nectar biochemistry of petunia is significantly different that or ornamental tobacco. First, petunia does not produce the high levels of hydrogen peroxide that are found in tobacco nectars and second, the nectar proteins found in petunia nectar are very different from those produced in tobacco nectars (Hillwig et al., 2010b). Because petunia and tobacco nectars are so very different, we initiated the current work is evaluate nectar biochemistry throughout the genus *Nicotiana*. We therefore have chosen a number of *Nicotiana* species that broadly represent the breadth of the *Nicotiana* to characterize their nectar production.

## Materials and Methods

### Materials

The materials used in these studies were obtained from either Fisher Chemical Co^1^. or Sigma Chemical Co^2^. and were of the highest quality available.

### Plant Materials

The tobacco species used for this study are shown in **Table 3**. Seeds were obtained from the United States Department of Agriculture National Genetic Resources Program^3^, plants were grown from seed in the greenhouse and when approximately 15 cm tall, these plants were transplanted to individual 30 cm pots containing a local potting mix. Plants were grown under 16 h day/8 h night conditions until flowering. Flowers were staged as described in (Koltunow et al., 1990).

### Floral Anatomy

To evaluate the floral morphology of these different *Nicotiana* sp., we characterized the size of the floral opening, where insects enter, the floral size, depth of the floral tube. Analysis of each of these features was characterized from at least 10 flowers each from three different tobacco plants.

Floral size was measured using a digital micrometer, placing one tine at the base of the flower and the other at the corolla-floral tube junction. Likewise, the floral opening was also measured using a digital micrometer, placing both tines at opposite faces of the corolla’s opening. The depth of the floral corolla was measured by inserting a short length of monofilament fishing line (30# test) until it stopped at the base of the gynoecium. The corolla-floral tube junction was then marked on the line, and after removing the line the depth of the corolla was measured.

### Carbohydrate Analyses

Nectar was collected from flowers, in the first hours of the day, as previously described (Carter et al., 1999; Naqvi et al., 2005). For quantitative analyses, 100 μL of raw nectar was collected and diluted (1:1000) using double distillated water. The samples were immediately returned to the laboratory for carbohydrate quantification. The levels of sucrose, glucose, and fructose were evaluated using the sucrose/D-glucose/D-fructose determination kit (Boehringer Mannheim/r-Biopharm^4^, catalog no. 10716260035), according to the manufacturer’s directions.

### Protein Quantification

Protein concentrations were determined by the dye-binding method described by (Bradford, 1976), with bovine serum albumin (BSA) as the standard.

### SDS-PAGE

Protein profiles from raw nectar were analyzed by SDS-PAGE according method described by (Laemmli, 1970).

### Hydrogen Peroxide in Nectar

Hydrogen peroxide in nectar was evaluated using the FOX reagent according by (Bleau et al., 1998; Hillwig et al., 2010b). Twenty-five microliters of nectar was added to 975 μL of distilled water. For analysis of H_2_O_2_ 200 μL of diluted nectar was used in the assay reaction with Fox reagent. The FOX reagent contained sulfuric acid 1.2 mM, xylenol orange 0.1 mM, ferrous ammonium sulfate 0.25 mM and sorbitol 0.1 mM. The H_2_O_2_ concentration in nectar was determined from a standard curve by measurement of the absorbance 560 nm.

### β-Carotene Analysis

To evaluate the levels of β-carotene in nectaries, we isolated 40 mg of nectary tissue from stage 12 flowers of each species. Care was taken to insure that non-nectary tissue was excluded from these samples as described (Horner et al., 2007). Carotenoids were extracted from the homogenate using two 1 mL aliquots of acetone followed by a 1 mL aliquot of hexane. The organic layers were combined, dehydrated with anhydrous sodium sulfate, evaporated to dryness, and taken up in 50-μL hexane for analysis. Carotenoid levels were estimated by absorption at 450 nm. The β-carotene was confirmed within each species by thin layer chromatography (TLC) on silica gel plates using an acetone: hexane (9:1) mobile solvent as previously described (Horner et al., 2007).

### Ascorbic Acid Analysis

The ascorbic acid analysis was performed according method described by Horner et al. (2007). For these analyses, either nectar was harvested from stage 12 flowers of each species. For analysis of raw nectar, 50 μL of nectar was added to 150 μL of distilled water. An aliquot of 50 μL was used for assay in a total volume of 2 ml of 1% oxalic acid. This was titrated to a pink endpoint with 0.05% 2,6-dichlorophenolindophenol (DCIP) in 0.1 M phosphate buffer, pH 7.0. A standard curve using ascorbate 0–20 μg of ascorbate was prepared to quantitate levels of ascorbate.

### Mass Spectrometry (LC-MS/MS)

The proteomic analysis was performed to identify proteins in nectars at the Iowa State University protein facility^5^. The nectar of different species was initially analyzed by SDS-PAGE according method described previously (Laemmli, 1970). Afterwards, the selected bands were excised and the pieces transferred to a 0.6 mL methanol, washed and then added 20 μl of 1% acetic acid. Next, the proteins were digested in solution with trypsin/Lys-C (Promega). The peptides were then separated by liquid chromatography and analyzed by MS/MS by fragmenting each peptide on Q Exactive^TM^ Hybrid Quadrupole-Orbitrap Mass Spectrometer from Thermo Scientific. Raw data were analyzed using Thermo Scientific’s Proteome Discoverer Software and the data searched using publically available databases. Bovine serum albumin was used as an internal calibration standard.

### Statistical Analysis

To perform the biochemical analysis we used three different tobacco plants and the floral nectar or nectaries collected from multiple flowers of each single plant to compose three independent biological replicates. The one way ANOVA test was performed to determine if there is a significant difference between mean of the each specie from the total and Tukey’s test, at *p* < 0.05 significance level, was used to analyze the differences between species. The statistical analysis was performed using the NCSS statistical software^6^.

## Results

From earlier studies, we observed that the nectar of ornamental tobacco differed significantly from the nectar of the closely related petunia (Hillwig et al., 2010a). To investigate this observation, we decided to examine the nectars from a variety of tobacco species across the genus *Nicotiana* to determine whether differences were observed within the tobacco genus. Because our earlier work was done with a species cross that fell within the section Alatae. Then based upon the phylogenetic studies of Bogani et al. (1997), Chase et al. (2003), and Clarkson et al. (2004) we selected five additional *Nicotiana* sections to enhance diversity within the genus *Nicotiana*. We obtained seed from numerous species of these sections from the United States Department of Agriculture National Genetic Resources Program^7^. These were grown to maturity and based upon growth characteristics as well as previously published analyses of pollinator preferences, we chose the *Nicotiana* species shown in **Table 1** for these analyses.

**Table 1:** *Nicotiana* species selected for these studies.

Number	Species	Section	Pollination syndrome	Reference
1	*N. rustica*	Paniculatae	Moth	Anon, Anon
2	*N. glauca*	Noctiflorae	Birds	Ollerton et al., 2012
3	*N. benthamiana*	Suaveolentes	Open, moth, bee, other	Anon, Anon
4	*N. clevelandii*	Polydicliae	Open, moth, bee, other	Anon, Anon
5	*N. sylvestris*	Petunoides	Hawkmoth	Mahr, 2013
6	*N. plumbaginifolia*	Alatae	Hawkmoth	Kaczorowski et al., 2005
7	*N. bonariensis*	Alatae	Small moth	Kaczorowski et al., 2005
8	*N. alata*	Alatae	Hawkmoth	Kaczorowski et al., 2005
9	*N. langsdorffii*	Alatae	Hummingbird, bee	Kaczorowski et al., 2005


Once the selected plant species were growing and flowering, we compared the floral characteristics of these species. For these values, we measured floral opening (throat width), the corolla length (floral base to corolla), and the depth of the floral corolla. Our interests were to determine the size of a pollinating insect that could enter the floral opening, as well as the depth of the corolla to determine the minimum length of the pollinator’s proboscis. This analysis shown in **Table 2** illustrate that there are different categories of flower size among our selected group. Long flowers (>7 cm) include the *N. alata* and *N. sylvestris* (*q*_s_ = 1.53, *p* < 0.899). The shortest flowers (<2.5 cm) include *N. rustica* (*q*_s_ = 33.22, *p* < 0.01) and *N. bonariensis* (*q*_s_ = 34.22, *p* < 0.01). The intermediate sized flowers ranged from (2.5 cm to 5 cm) encompass the remainder *N. benthamiana* (*q*_s_ = 19.42, *p* < 0.01), *N. plumbaginifolia* (*q*_s_ = 20.44, *p* < 0.01), *N. glauca* (*q*_s_ = 22.49, *p* < 0.01), *N. clevelandii* (*q*_s_ = 25.55, *p* < 0.01), and *N. langsdorffii* (*q*_s_ = 29.22, *p* < 0.01). We also determined the depth of the corolla and we found that the ratio of the corolla depth to the flower size was different, from 68% in *N. glauca* to 90% in *N. alata*. Thus, we found that the length of the floral tube is indicative of the length of the pollinator’s proboscis required to reach any nectar at the base of the flower. Also shown in **Table 1** is the pollinator syndrome that is used by these species. In species with long flowers (*N. alata* and *N. sylvestris*) are preferred by hawkmoth, while species with intermediate or short size flowers (*N. glauca* or *N. langsdorffii*) are preferred by bird, hummingbird or bee.

**Table 2:** Floral characteristics among the selected *Nicotiana* sp. dimensions were determined as outlined in the Section “Materials and Methods.”

Species	Floral opening (mm)	Floral size (cm)	Corolla depth (cm)	Ratio (Cd/Fs) × 100%
*N. rustica*	5.1 ± 0.7^c^	1.6 ± 0.1^e^	1.3 ± 0.2^d^	81%
*N. glauca*	4.9 ± 0.0^b^	3.7 ± 0.1^bc^	2.5 ± 0.1^c^	68%
*N. benthamiana*	2.8 ± 0.3^c^	4.3 ± 0.5^b^	3.6 ± 0.5^b^	84%
*N. clevelandii*	3.6 ± 0.5^c^	3.1 ± 0.2^cd^	2.2 ± 0.2^c^	71%
*N. sylvestris*	2.9 ± 0.3^c^	7.8 ± 0.6^a^	6.9 ± 0.6^a^	88%
*N. plumbaginifolia*	3.1 ± 0.3^c^	4.1 ± 0.3^b^	3.2 ± 0.2^b^	78%
*N. bonariensis*	2.9 ± 0.2^c^	1.4 ± 0.1^e^	1.0 ± 0.1^d^	71%
*N. alata*	7.9 ± 0.5^a^	8.1 ± 0.5^a^	7.3 ± 0.5^a^	90%
*N. langsdorffii*	5.7 ± 0.5^b^	2.4 ± 0.2^de^	1.7 ± 0.1^d^	71%


*Different letters mean significant differences between species within the same characteristics at p < 0.05 for significance level*.

### Carbohydrate in Nectar

**FIGURE 1:**
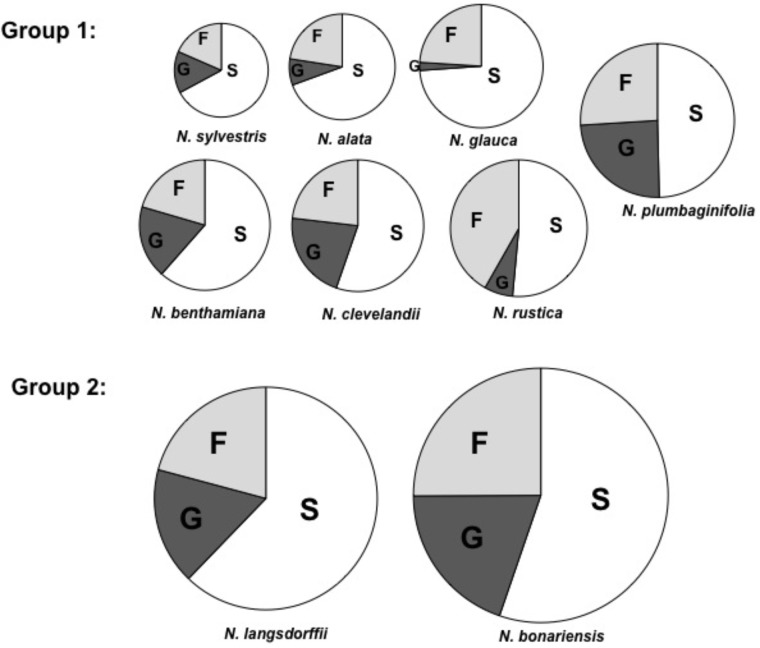
Carbohydrate composition of selected tobacco species. Proportions of fructose (F), glucose (G), and sucrose (S) in nectar. The area of each circle represents the total mass of sugar found in each nectar sample. Each segment represents the mean of three samples. Averages ± standard deviation (*N* = 3). All characteristics of each nectar are present in **Table 3**. Group 1, total nectar carbohydrate < 560 mM, Group 2, total nectar carbohydrate > 1000 mM.

To begin analysis of nectar from these species, we examined the nectar carbohydrate from each of the selected plant species. Using a Sucrose/Glucose/Fructose analysis kit from Boehringer Mannheim/r-Biopharm, we measured each of these components and determined the molar ratios of each of these sugars in the different nectars (**Table 3**). These carbohydrate composition data (**Figure 1**) show two different groups: Group 1 – *N. glauca, N. benthamiana, N. clevelandii, N. alata, N. sylvestris, N. rustica*, and *N. plumbaginifolia* composed mainly of night flowering *Nicotiana* species showed the lower levels of sugars (<560 mM), while that day flowering *Nicotiana* species *N. bonariensis* and *N. langsdorffii*, showed the higher levels of sugars content (>1000 mM ). For most of these species such as, *N. benthamiana, N. clevelandii, N. sylvestris*, and *N. plumbaginifolia*, the molar ratio of Glucose to Fructose was very similar. However, for a few species, notably the day flowering *Nicotiana* species, *N. glauca* and *N. rustica*, there was significantly more Fructose than Glucose. Similar observations have been made for these species (Tiedge and Lohaus, 2017), and recent findings Tiedge and Lohaus (2017) suggest that the differences in nectar sugars composition may be implicated with different mechanisms of secretion between day/night flowering *Nicotiana* species.

**Table 3:** Free sugars composition in nectar of different *Nicotiana* species.

Species	Sugar Concentration (mM)				Sugar (%)			Molar ratio			Ratio	
			
	*S*	*G*	*F*	Total	*S*	*G*	*F*	*S*	*G*	*F*	*F*/*G*	*S*/(*G*+*F*)
*N. glauca*	244 ± 2^d^	7 ± 2^h^	79 ± 2^f^	330 ± 3^g^	24 ± 1	1 ± 0	8 ± 3	0.7	0.0	0.4	11.0	1.8
*N. benthamiana*	235 ± 7^d^	68 ± 3^e^	78 ± 1^f^	381 ± 1^f^	23 ± 1	7 ± 1	8 ± 1	0.7	0.4	0.4	1.0	0.9
*N. clevelandii*	226 ± 5^d^	88 ± 2^d^	95 ± 2^e^	409 ± 3^e^	22 ± 3	9 ± 2	10 ± 1	0.7	0.5	0.5	1.0	0.7
*N. sylvestris*	148 ± 3^f^	31 ± 7^f^	40 ± 9^h^	219 ± 5^i^	15 ± 2	3 ± 1	4 ± 1	0.4	0.2	0.2	1.0	1.0
*N. alata*	194 ± 5^e^	22 ± 1^f^	63 ± 2^g^	279 ± 3^h^	19 ± 2	2 ± 1	6 ± 1	0.6	0.1	0.3	3.0	1.5
*N. plumbaginifolia*	286 ± 2^c^	141 ± 2^c^	150 ± 1^d^	577 ± 2^c^	28 ± 2	14 ± 1	15 ± 1	0.8	0.8	0.8	1.0	0.5
*N. rustica*	236 ± 4^d^	31 ± 3^f^	191 ± 3^c^	458 ± 4^d^	23 ± 1	3 ± 1	19 ± 1	0.7	0.1	1.1	10.0	0.6
*N. langsdorffii*	715 ± 1^b^	195 ± 3^b^	239 ± 7^b^	1149 ± 1^b^	71 ± 1	20 ± 1	24 ± 1	2.1	1.1	1.3	1.2	0.9
*N. bonariensis*	794 ± 4^a^	282 ± 4^a^	360 ± 1^a^	1436 ± 1^a^	79 ± 1	28 ± 1	36 ± 1	2.3	1.6	2.0	1.3	0.6


*Different letters mean significant differences between species within the same characteristics at p < 0.05 for significance level.*

### Nectary Carotenoids

After analyzing the floral characteristics and the carbohydrate composition of the nectars, we then examined the gynoecium and nectary gland of each of these species. Because of our interest in the development of the floral nectary during the process of floral growth, we examined the gynoecia of these species at four different floral stages: Stage 6 (pre secretion), Stage 9 (at the time of the metabolic switch, (Ren et al., 2007b), Stage 12 (anthesis, with full nectar secretion) and the Post-fertilization Stage (48 h after pollination). These stages are shown in Supplementary Figures S1–S4. In all cases, the gynoecium and nectary gland from each species increase in size and the color changes from light yellow or lime green at the earliest stages to a bright orange in nectaries of mature stages. The observed changes were very similar to the development of the nectaries of the interspecific cross LxS8 (Horner et al., 2007). Based upon the obvious swelling of the nectaries and the noticeable color changes, we hypothesized that similar developmental pathways (involving carotenoid accumulation (Horner et al., 2007) and starch buildup and breakdown (Ren et al., 2007b) likely exist in these different *Nicotiana* species. One striking feature that we observed was extreme levels of carotenoids that were present in the nectaries of *N. bonariensis*. This is shown best by comparing the color of *N. bonariensis* (Supplementary Figure S3, #15 and #24) with similarly staged nectaries of the other species in Supplementary Figure S3.

**FIGURE 2:**
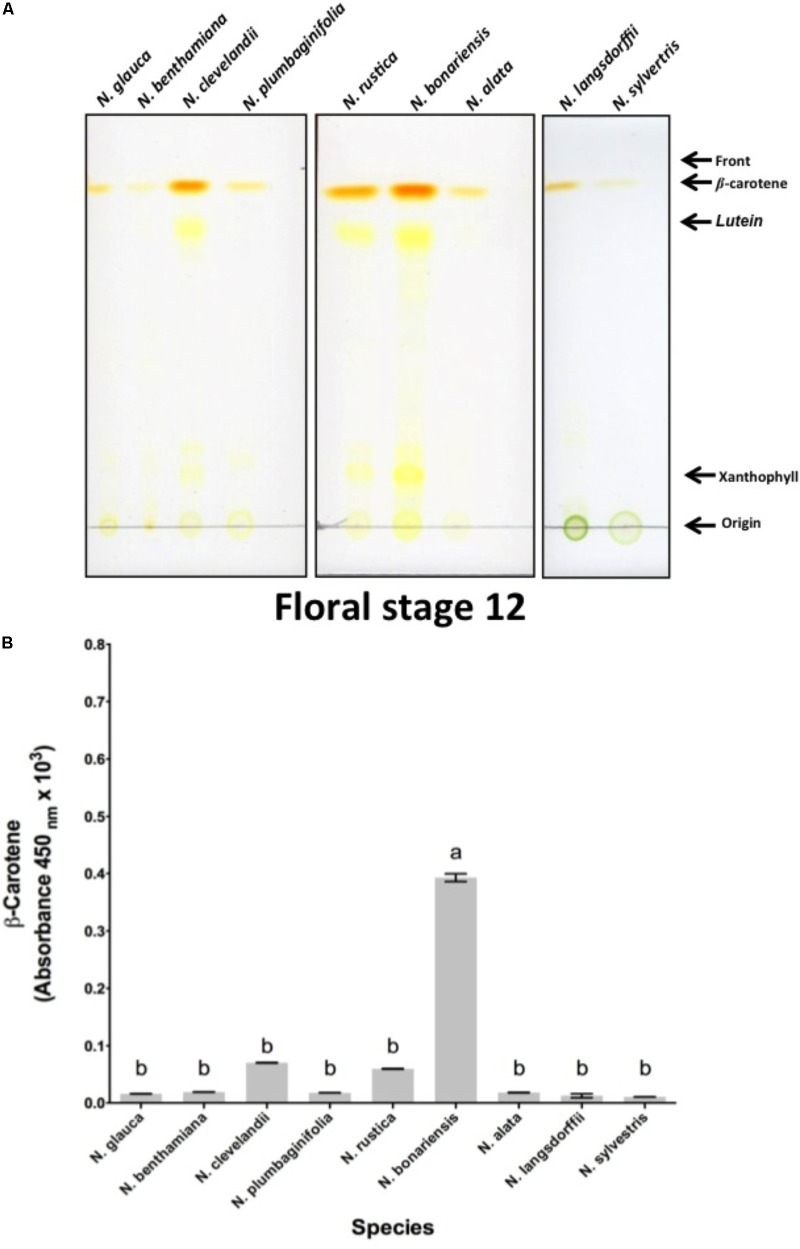
Analysis of nectary-expressed carotenoids from different species of *Nicotiana* sp. **(A)** Silica gel thin-layer chromatogram (TLC) of carotenoids isolated from stage 12 nectaries of *N. glauca*; *N. benthamiana*; *N. clevelandii*; *N. plumbaginifolia*; *N. rustica*; *N. bonariensis* and *N. alata; N. langsdorffii;* and *N. sylvestris.* The origin, solvent front, and migration of various pigments are indicated. Solvent used was 9:1 acetone: hexane solvent. **(B)** Absorbance (450 nm) of β-carotene in nectary extracts at stage 12. Averages ± standard deviation (*N* = 3). Species evaluated: 1, *N. glauca*; 2, *N. benthamiana*; 3, *N. clevelandii*; 4, *N. plumbaginifolia*; 5, *N. rustica*; 6, *N. bonariensis;* 7, *N. alata;* 8, *N. langsdorffii;* and 9, *N. sylvestris.* Different letters mean statistical differences between groups.

To confirm our hypothesis that similar processes were occurring in the nectaries of these different species, we investigated the biochemistry of these different nectary glands. In LxS8, the orange color arises from β-carotene that is produced from isopentenyl pyrophosphate (IPP) arising from the dimethylallyl pyrophosphate (DMAPP) pathway (Horner et al., 2007). Therefore, we examined the level of β-carotene that was present in the Stage 12 floral nectaries of each species. As shown in **Figure 2A**, a bright orange pigment that co-chromatographed with β-carotene (*R*_f_ = 0.95) was present in the nectaries of each of the *Nicotiana* species. The amount of β-carotene varied significantly between *N. bonariensis* and other species showing the highest levels. Other species such as *N. sylvestris, N. benthamiana*, and *N. glauca* showed much lower levels of β-carotene. Note that several intermediate pigments that were also yellow were also observed. These were identified as lutein and xanthophyll by virtue of their *R*_f_s = 0.80 and 0.17 (Schoefs, 2005). For each of the selected species, we further spectroscopically quantified the level of β-carotene. The results shown in **Figure 2B**, mirror the levels that were chromatographically identified in **Figure 2A**. In this analysis, *N. bonariensis* showed the highest levels of β-carotene (*p* < 0.001, df = 17, *n* = 18), confirming the observations of nectaries shown in the Supplementary Figure S3.

In addition to the presence of β-carotene in the nectary gland, the LxS8 interspecific cross also showed an additional antioxidant present in soluble nectar, ascorbate (Carter and Thornburg, 2004b). To determine whether these *Nicotiana* species also express this important nectar antioxidant, we evaluated whether ascorbate was present in the nectar of these selected species. The highest levels ascorbate was evidenced in *N. alata* and varied significantly (*p* < 0.001, df = 25, *n* = 27) of the other species, while, *N. langsdorffii* and *N. sylvestris* showed the lower levels, **Figure 6**.

### Hydrogen Peroxide in Nectar

**FIGURE 3:**
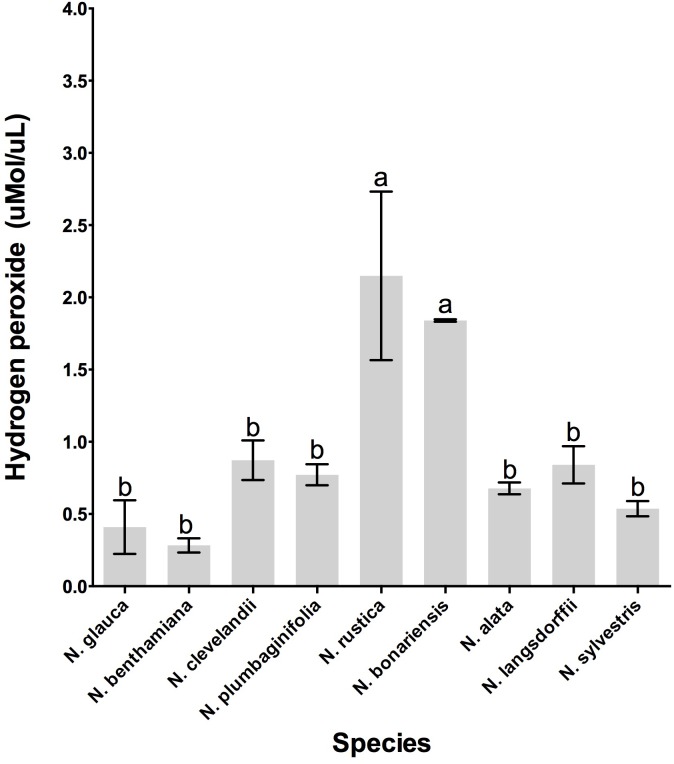
Hydrogen peroxide production in the floral nectar of the selected *Nicotiana* species. Averages ± standard deviation (*N* = 3). Species evaluated: 1, *N. glauca*; 2, *N. benthamiana*; 3, *N. clevelandii*; 4, *N. plumbaginifolia*; 5, *N. rustica*; 6, *N. bonariensis;* 7, *N. alata;* 8, *N. langsdorffii*; and 9, *N. sylvestris.* Different letters mean statistical differences between groups.

Previous analyses have demonstrated that LxS8 tobacco nectar had high levels of hydrogen peroxide (Carter et al., 1999). To determine whether other *Nicotiana* species also showed high levels of hydrogen peroxide, nectars were collected and their hydrogen peroxide content were measured with the FOX reagent method as described in Section “Materials and Methods.” As shown in **Figure 3**, the nectar of all species do indeed contain hydrogen peroxide. However, two species, *N. rustica* and *N. bonariensis* had very high levels of hydrogen peroxide, 2.14 and 1.84 μmol.ml^-1^, respectively. This could correlate with the high levels of sugars that were found in these species, especially in *N. bonariensis*. The high levels of sugar demonstrated in these species (**Table 3**) could increase the attractiveness of pollinators, having easier access to the nectar due to the floral characteristics (**Table 2**) increasing the colonization by microorganisms. The high levels of hydrogen peroxide would be a mechanism of control of microorganisms in nectar. In the other species, we found that the level of hydrogen peroxide was lower. Reasons for this are unclear, but may be related by altered regulation between the species in these complex pathways.

### Proteins in Nectar

**FIGURE 4:**
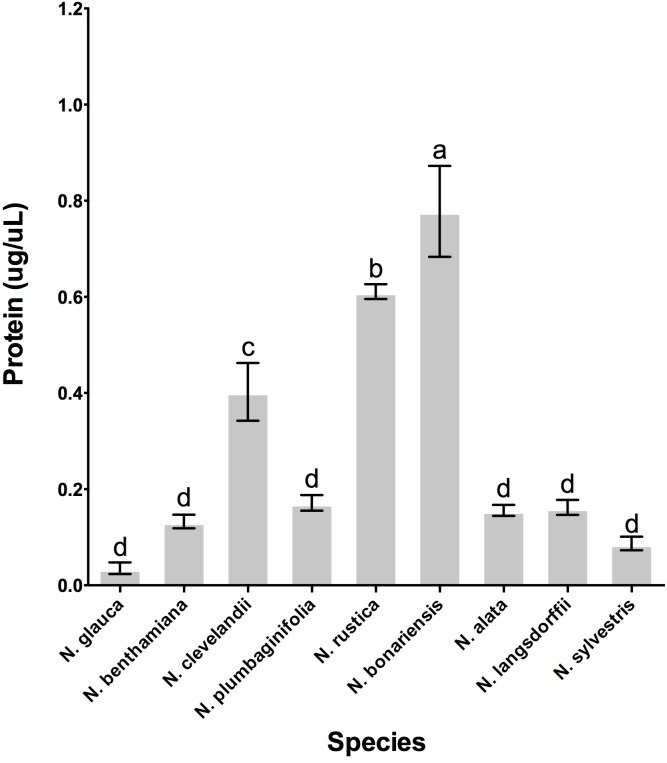
Protein accumulation in the nectar of various *Nicotiana* sp. Species evaluated: 1, *N. glauca*; 2, *N. benthamiana*; 3, *N. clevelandii*; 4, *N. plumbaginifolia*; 5, *N. rustica*; 6, *N. bonariensis;* 7, *N. alata;* 8, *N. langsdorffii;* and 9, *N. sylvestris.* Different letters mean statistical differences between groups.

To determine whether the different *Nicotiana* species also showed the presence of proteins in their nectars, we quantitated the amount of protein present in the nectars from each of these species. As shown in **Figure 4** the variability of nectar proteins was quite large, with some species such as *N. glauca* and *N. sylvestris* having very little protein in their nectars (0.044 μg protein/μL of nectar) while other species such as *N. clevelandii, N. rustica*, and *N. bonariensis* containing higher concentrations of protein in their nectars (up to 0.778 μg protein/μL of nectar).

**FIGURE 5:**
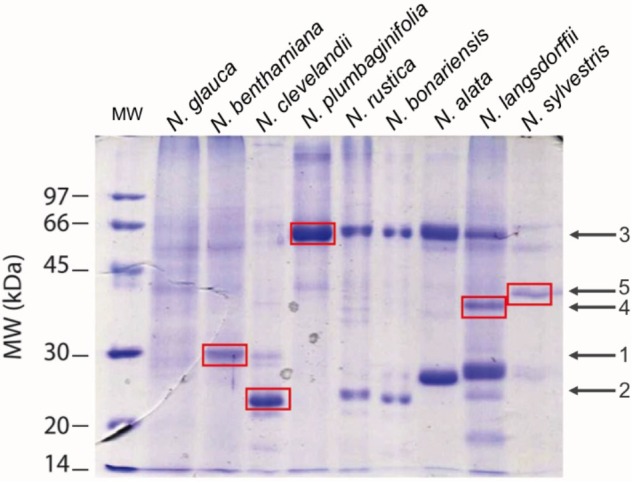
Patterns of nectar proteins by SDS-PAGE 12.5%. In each lane was applied 1.25 μgP of raw nectar. Averages ± standard deviation. The standard profile was obtained from the analysis of three different independent experiments (*N* = 3). The proteins were stained with Coomassie Blue. Averages ± standard deviation (*N* = 3).

Once we had confirmed that these species do indeed contain nectar proteins, we next wanted to identify the nectar proteins in these different species. First we investigated the profile of proteins in nectars among the species. SDS-PAGE analysis showed different profiles of the proteins in nectars **Figure 5** distributed between 70 and 20 kDa. The protein profile of *N. rustica, N. bonariensis, N alata*, and *N. langsdorffii* were very similar to the protein profile observed in the LxS8 interspecific cross (Carter and Thornburg, 2004a) and suggested that the nectarins found in LxS8 may also accumulate in nectar of these other *Nicotiana* species. The protein quantification also varied significantly (*p* < 0.001, df = 17, *n* = 27) among the species.

Based upon the SDS PAGE protein gels, it initially appears that there are many different proteins present in these different *Nicotiana* species. Therefore, we excised five of these proteins from the gel (identified by red spots) and following trypsin digestion; we subjected them to proteomic analysis. The results of this analysis is shown in **Table 4** and in detail in Supplementary Figures S5A–C. The spots 1 (from *N. benthamiana*) and 2 (from *N. clevelandii*) shown in **Table 4**, were identified as Nectarin 1-like superoxide dismutases although these proteins had showed differences between the predicted molecular mass by SDS-PAGE 28 and 23 kDa, and the mass found by mass spectrometry 24.6 kDa. The different molecular weight likely is due the degree of glycosylation of Nectarin 1 proteins as was showed previously by Carter et al. (1999).

**Table 4:** Proteins from *Nicotiana* species nectars by ESI-Q-TOF MS/MS.

Spot No.	Theoretical		^∗^PEP Score	^∗∗^PSM	Identified peptides	Coverage (%)	Accession	Protein description
							
	MW (kDa)	pI						
#1	24.6	6.54	28.89	515	KVNGFPCKTNFTA	24.89	Q94EG3	Nectarin-1
					HSKVKVNGFPCKT			
					HPRASEMVFVMEG			
					SEMVFVMEGELDV			
#2	24.6	6.54	5.59	42	IDYAPGGINPPHTHPR	6.98	Q94EG3	Nectarin-1
#3	59.8	8.60	15.93	329	KSMEEDLFWAIR	2.25	Q9SA89	Berberine bridge like enzyme (Nectarin-5)
#4	31.5	6.74	120.3	910	LVHESNNGKFVVI	55.47	Q84UV8	Bifunctional monodehydro ascorbate reductase and carbonic anhydrase (Nectarin-3)
					HLVHESNNGKFVV			
					YDEKSENGPANWG			
					SENGPANWGNIRP			
					GPANWGNIRPDWK			
					RPDWKECSGKLQS			
					PSEHTINGERFNL			
					TQYQLKQLHWHTP			
					SLTTPPCTEGVVW			
					HDGFETNARPTQP			
					PDPFLSMIENDLK			
					TNARPTQPENERY			
					RPTQPENERYINS			
					RQIKLLQEAVHDG			
#5	31.5	6.74	28.30	35	LVHESNNGKFVVI	28.30	Q84UV8	Bifunctional monodehydro ascorbate reductase and carbonic anhydrase (Nectarin-3)
					HLVHESNNGKFVV			
					SLTTPPCTEGVVW			


*^∗^PEP score: Measures the significance of a single spectrum assignment with a specific PSM score. It is the probability of the PSM being incorrect, i.e., PEP of 0.01 means there is a 1% chance of the PSM being incorrect. ^∗∗^PSM: peptide-spectrum match, a spectrum that matches to a peptide sequence.*

Furthermore, the data suggest that Nectarin 1 is one of the main proteins found in the nectar of *N. benthamiana* and *N. clevelandii.* The major protein identified in nectar of *N. plumbaginifolia* was identified as Nectarin 5, spot 3, **Table 4**. The theoretical molecular weight was 59.8, very similar to obtained by SDS PAGE 60 kDa. The spots 4 from *N. langsdorffii* and 5 from *N. sylvestris* were identified as a Nectarin 3-like protein.

## Discussion

Because of previously observed significant differences between the nectars of the genus *Nicotiana* (Carter and Thornburg, 2004a) and the genus petunia (Hillwig et al., 2010a), we have investigated the nectars of a broad group of *Nicotiana* species to determine whether significant differences in nectars exist within this genus. To attract their pollinators, the plants offer floral nectar secreted into the floral tube at the base of the ovary that constitute a rich source of sugars, amino acids, vitamins and other ingredients which provides a rich reward to pollinators (Carter et al., 2006). However, the selected species used in this study include several different pollinator syndromes, **Table 1**.

It is known that several factors such as sugar composition, amino acids, organic acids and inorganic ions can affect the visitation of pollinators (Kessler and Baldwin, 2007; Afik et al., 2014; Tiedge and Lohaus, 2017). In addition, another important aspect as the floral biology can affect the access of the pollinators (Ackermann and Weigend, 2006). Thus, we conducted a study to understand the relationship between floral biology and the biochemistry of nectar from different genus of *Nicotiana*. As shown in **Table 2**, species like *N. benthamiana, N. clevelandii N. plumbaginifolia* showed intermediate flowers or in other species such as *N. alata* and *N. sylvestris* long flowers. Due to the floral characteristics these species have access to nectar more limited requiring specialized pollinators with long proboscis like hawkmoth. On the other hand, species like *N. rustica, N. bonariensis*, and *N. langsdorffii* showed short flowers indicating that nectar can be more easily accessed and has different composition. The nectar sugar concentration also differed among *Nicotiana* species, being divided into two groups. The sugars were higher in species with short flowers such as *N. bonariensis* and *N. langsdorffii*, while that other species showed lower concentrations (**Figure 1**). In species with intermediate or long flowers, there was no observed correlation between the floral length and the concentration of sugar. Recently, (Tiedge and Lohaus, 2017) showed that this correlation is associated with the floral opening period. Day flowering *Nicotiana* species such as, *N. rustica and N. langsdorffii* show higher level of sugar than night flowering *Nicotiana* species. In addition of the floral biology, the nectar sugar composition is another factor that can significantly affect the visitation of pollinators (Torres and Galetto, 2002; Wolff, 2006; Witt et al., 2013). Sucrose represents one of the main sugars found in nectar (Chalcoff et al., 2006). The analysis of nectar carbohydrate composition from *Nicotiana* species **Table 3** showed that sucrose was the major sugar in floral nectar in all species analyzed. Three species, *N. alata, N. glauca*, and *N. sylvestris*, showed molar ratio (S/G + F) ≥ 1.00. From these species only *N. glauca* having intermediate-length flowers has been described as diurnal flowering species, while *N. alata* and *N. sylvestris* are night-flowering species. The higher sucrose to hexose molar ratio was previously shown in nectar of long night-flowering *N. alata* and *N. sylvestris* and common feature of night *Nicotiana* flowering species (Tiedge and Lohaus, 2017). The high content of sucrose in nectar of night-flowers or with long floral tubes is associated with higher starch storage in nectaries and different mechanisms of nectar secretion (Tiedge and Lohaus, 2017). Furthermore, the high content of sucrose is related to decrease in viscosity, which facilitates suction by pollinators with long proboscis (Nicolson et al., 2013; Tiedge and Lohaus, 2017). In addition, during the night with lower temperatures, the evaporation effect is reduced and is not necessary high osmolarity for nectar secretion. Thus, long flowered plants takes advantage of these conditions to secrete sucrose, a carbohydrate with low osmolarity (Witt et al., 2013). In addition to sucrose, the nectar of *Nicotiana* species also presented glucose and fructose in their composition and among the hexoses analyzed, fructose was the predominant sugar (**Table 3**). In some *Nicotiana* species studied, the fructose/glucose (F/G) molar ratio was higher or equal 1.0. However, an extremely high molar ratio (F/G) of 11.0 was observed in nectar of *N. glauca*, followed by *N. rustica*, with molar ratio of 10.0 (**Table 3**). Recently a high molar ratio (F/G) of 12.6 for nectar of *N. glauca* was described, suggesting that this feature is characteristic of this species. The high content of fructose in nectars has been associated with increase sweetness, thus increasing pollinator reward (Tiedge and Lohaus, 2017). Besides the floral characteristics and carbohydrate composition of the nectars, we also examined the biochemistry of nectary gland. In all species, the nectary gland increased in size and changed color as result of carotenoids accumulation Supplementary Figures S1–S4. Carotenoids (b-carotene) were observed in nectaries, and extreme levels were observed in nectaries of *N. bonariensis* **Figures 2A,B**. In nectaries, the production of carotenoids and ascorbate provides an antioxidant defense against the high level of hydrogen peroxide found in nectar (Horner et al., 2007). During development of nectaries, the high level accumulation of carotenoids in the nectaries starts about stage 9 when nectaries undergo a metabolic shift and starch are degraded to produce glucose. This glucose is then available to the methylerythritol phosphate (MEP) pathway, which leads to the production of IPP, the carotenoid precursor. The high levels of carotenoids are thought protect nectary cells from the severe oxidative processes that occur as a result of the Nectar redox cycle (Carter and Thornburg, 2004c).

**FIGURE 6:**
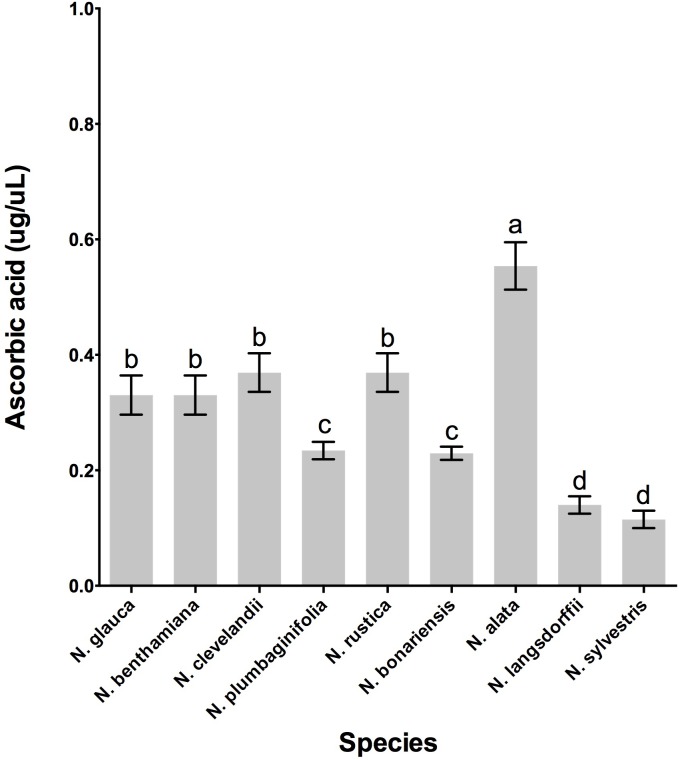
Quantity of ascorbate from nectar of nine tobacco species flowers at stage 12. Averages ± standard deviation (*N* = 3). Species evaluated: 1, *N. glauca*; 2, *N. benthamiana*; 3, *N. clevelandii*; 4, *N. plumbaginifolia*; 5, *N. rustica*; 6, *N. bonariensis;* 7, *N. alata;* 8, *N. langsdorffii;* and 9, *N. sylvestris.* Different letters mean statistical differences between groups.

In fact, the nectar of *N. bonariensis* showed one of the highest content of sugars and hydrogen peroxide (**Figure 3**). Thus, the high levels of carotenoids and other pigments such as lutein and xanthophyll may function as an additional defense to high level of hydrogen peroxide in nectar of this species. Similar observation was found in nectar of *N. rustica*, species with short flowers. In *N. rustica*, the hydrogen peroxide content showed the highest levels among all species tested (**Figure 3**), however, there appeared to be no correlation with the high levels of carotenoids or ascorbate. Although, *N. rustica* has intermediate sugar content, the short size flowers could facilitate the access pollinators and growth of microorganisms. Thus, the highest hydrogen peroxide content would be an additional nectar defense. The ascorbate is another important antioxidant involved in the Nectar Redox Cycle. Ascorbate was detected in nectar of all species, however, *N. alata* showed the highest levels (**Figure 6**). Ascorbate accumulates at high levels in nectaries at stage 12 (2 μg/nectary) (Horner et al., 2007), composing the nectar during the secretion process and integrating the Nectar Redox Cycle. As previously described, the Nectar Redox Cycle is the remarkable biochemistry pathway responsible for production of high levels of hydrogen peroxide in nectar. The SDS-PAGE analysis showed different profiles of the proteins in nectars **Figure 6**. *N. plumbaginifolia, N. langsdorffii, N. bonariensis*, and *N. alata* from Alatae section had similar profile. The proteomics analysis of the main protein in nectar of *N. plumbaginifolia* identified as Nectarin 5, spot 3, **Table 4**, being this protein was very evident Alatae section. This is indicative that Nectarin 5 has a central role in the production of peroxide in Alatae section. Other species such as *N. glauca* and *N. sylvestris* had very little protein in their nectars, low abundance without majority proteins. In ornamental tobacco nectar, the nectarins are secreted as array of five proteins and accumulate to almost 250 mg/ml in nectar (Carter et al., 1999). The low abundance of proteins in the nectar *N. glauca, N. sylvestris*, and *N. benthamiana* may be associated the low content of hydrogen peroxide quantified these nectars. The very limited production of hydrogen peroxide suggesting that a different mechanism may exist for antimicrobial defense, as RNase activities described to petunia nectar (Hillwig et al., 2010a). Based upon these observations, we conclude that, although the oxidative processes that were first identified and characterized in the LxS8 interspecific cross, including the presence of hydrogen peroxide in nectar as well as antioxidants in both soluble nectar (ascorbate) and in nectary tissues (β-carotene) have been identified in all species, there are species-specific differences are found throughout the genus *Nicotiana*. Further, the major nectar proteins that we identified from these species belonged to the nectarin family of proteins (especially, Nec1, Nec3, and Nec5).

## Author Contributions

FS and RT designed the study, performed the biochemical assays, interpreted the experimental data, and wrote the manuscript. AG cultivated the plants and collected the samples used in all experiments. All authors read and approved the final version of the manuscript.

## Conflict of Interest Statement

The authors declare that the research was conducted in the absence of any commercial or financial relationships that could be construed as a potential conflict of interest.

## Footnotes

1 www.fishersci.com

2 www.sigmaaldrich.com

3 http://www.ars-grin.gov

4 www.r-biopharm.com

5 www.protein.iastate.edu

6 www.ncss.com

7 www.ars-grin.gov

## Abbreviations

EST: expressed sequence tagged mRNA
Floral Nectary stages
S6: Stage 6 (immature; beginning of metabolic switch)
S9: Stage 9 (immature; pre-secretory
S12: Stage 12 (mature; anthesis)
PF: post-fertilization
LxS8: an interspecific cross of *Nicotiana langsdorffii* × *N. sanderae* long studied for nectar/nectary research
