# Supplementary material for: Nectar Analysis Throughout the Genus Nicotiana Suggests Conserved Mechanisms of Nectar Production and Biochemical Action
Source: Front Plant Sci. 2018 Jul 30;9:1100. doi: 10.3389/fpls.2018.01100 (PMC6077755; doi:10.3389/fpls.2018.01100)
Supplement: Supplementary file 4 [file Image_4.PDF]

Supplemental Figure 4
Post fertilization:

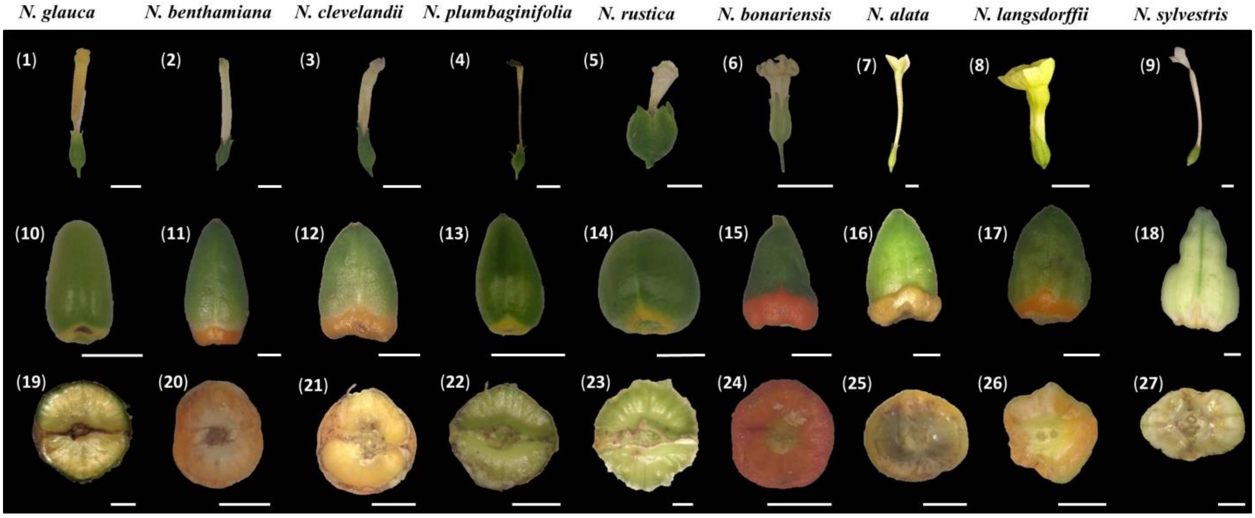

Supplemental Figure 4. Flowers (**1-9**), ovary (**10-18**) and nectary gland cross section (**19-27**) of nine species from *Nicotiana at* post fertilization. (**1**, **10** and **19**) *N. glauca*; (**2**, **11** and **20**) *N. benthamiana*; (**3**, **12** and **21**) *N. clevelandii*; (**4**, **13** and **22**) *N. plumbaginifolia*; (**5**, **14** and **23**) *N. rustica*; (**6**, **15** and **24**) *N. bonariensis;* (**7**, **16** and **25**) *N. alata;* (**8**, **17** and **26**) *N. langsdorffii;* (**9**, **18** and **27**) *N. sylvestris* (**1-9** Bar = 1 cm); (**10-18** Bar = 0.1 cm); (**19-27** Bar = 0.1 cm).
