# Supplementary material for: Nectar Analysis Throughout the Genus Nicotiana Suggests Conserved Mechanisms of Nectar Production and Biochemical Action
Source: Front Plant Sci. 2018 Jul 30;9:1100. doi: 10.3389/fpls.2018.01100 (PMC6077755; doi:10.3389/fpls.2018.01100)
Supplement: Supplementary file 5 [file Image_5.PDF]

Supplemental Figure 5A. Identification of proteins from peptides in spots 1 and 2 (see Figure 6).

UniProt Accession Q94EG3 (LxS8 Nectarin-1)

```
                    10         20         30         40         50
Q94EG3      MAAFGINSKI FQSMEMAILF LLAISIDRYC FAADEDMLQD VCVADLHSKV
Peptide 1                                                     HSKV

                    60         70         80         90        100
Q94EG3      KVNGFPCKTN FTAADFSSLA ISKPGATNNK FGSVVTTANV EQVPGLNTLG
Peptide 1a  KVNGFPCKT
Peptide 1b  KVNGFPCKTN FTA

                   110        120        130        140        150
Q94EG3      VSLARIDYAP GGINPPHTHP RASEMVFVME GELDVGFITT ANVLVSKKII
Peptide 1c                    HP RASEMVFVME G
Peptide 1d                         SEMVFVME GELDV
Peptide 2a       IDYAP GGINPPHTHP R

                   160        170        180        190        200
Q94EG3      KGEVFVFPRG LVHFQKNNGE VPAAVISAFN SQLPGTQSIP ITLFGASPPV

                   210        220
Q94EG3      PDDVLAQTFQ INTEDVQQIK SKFAPVKKF
```

Peptides 1a – 1d and 2a were identified via mass spectrometry and are listed in Table 4.

Supplemental Figure 5B. Identification of proteins from peptides in spot 3 (see Figure 6).

UniProt Accession Q84N21 (LxS8 Nectarin-5)
UniProt Accession Q9SA89 (*Arabidopsis thaliana* Berberine bridge enzyme-like 12)

```
Q84N21          MTMSLLSYLSLLIFLSSLCAASVDVQKKFLQCLS------VSDQKFPIYTTNNKNYSSVL
Q9SA89          MYLIFLLFFAASYSMSLSSADSVTIYEDFVQCFKNVTTISDIDLSDVVLPRTSISFTPTL
                * : :* :::    :*  .* ** : :.*:**:.        * .  : . .. .::..*

Q84N21          QFSIQNLRFNTTKTPKPLVIVTPVSEAEIQRVILCAKESSIHVRVRSGGHDYEGLSYVSE
Q9SA89          RAYIRNARFNTSSMPKPSIIIVPRVDSHVQAAVICAKTLNLQLKIRSGGHDYDGLSYVSA
                :  *:* ****:. *** :*:.*  ::.:* .::***  .:::::*******:******

Q84N21          DPFVLIDLVGHRNITINVDDK--TAWVETGSTIGELYYKISKKSKTLGFPAGLCPTVGVG
Q9SA89          VTFLVLDLSNFRNITVDLNDGGGSAWVQTGATLGELYYRIWEKSEVHAFPAGVCPTVGVG
                 .*:::** ..****::::*   :***:**:*:*****:* :**:. .****:*******

Q84N21          GHISGGGTGVMLRKYGLAADNVIDARLMDANGRILDRKSMGEDLFWAIRGGGGNTFGLVL
Q9SA89          GHVSGGGYGHMIRKFGLTIDHVVDATIVDANGQIHDRKSMEEDLFWAIRGGGGGSFGVVL
                **:**** * *:**:**: *:*:** ::****:* ***** ************.:**:**
Peptide 3a                                           KSMEEDLFWAIR

Q84N21          AWKIKLVDVPEKVIVFTIDKTLEQNATKLVHKWQYVSSKLHQDLYIRIFIHKDEQNIFLA
Q9SA89          AFKVKLVTVPKTVTVFRVDKSVDENALDMVYKWQFVAPRTDPGLFMRVLLSSPTQNKTST
                *:*:*** **:.* ** :**::::** .:*:***:*:.: . .*::*::: .  **   :

Q84N21          S---FVSIFLGDIDRLLLIMQENFPELGLVRENCIEMSWIESTLYFAGFPRGESLD--VL
Q9SA89          VNTKLRALYLGKADDVVLKMAEEFPELGLKKEDCKEMTWIQSLLWWMNHVDVDKVKPEIL
                    : :::**. * ::* * *:****** :*:* **:**:* *:: ..   :.:.  :*

Q84N21          RSRGLPPTLYSEAKADYVQKPISVQQLEGIWDFFNAGEAKFEQMIFTPYGGRMDEISEYE
Q9SA89          LEREPDSAKFLKRKSDYVEKEMTKPELN--RLFQKLATLDRTGLVLNPYGGSLNVTAVNA
                 .*   .: : : *:***:* ::  :*:    * : .  .   :::.**** ::  :

Q84N21          LPFPHRPGNLYEIQYLMFWDEEGVEEAERHMRWMRRLYAHMEPLVSTSPRAAYINYRDLD
Q9SA89          TAFPHR-HKLYKIQHSVTWPDAGPEAERLYIGNLRTTYNIMTPFVSKNPRSSYLNYRDID
                 .****  :**:**: : * : * *  . ::  :*  *  * *:**..**::*:****:*

Q84N21          IGVNNKKGNTSYAQAKVWGIKYFKNNFDRLVQVKTKVDPSNVFRNEQSIPPLVEQE-
Q9SA89          IGVN-DHGADGYRKGEIYGRKYFGENFDRLVRVKTAVDPDNFFRNEQSIPTLPPNRR
                **** .:*  .* :.:::* *** :******:*** ***.*.********.*  :.
```

Peptide 3a was identified via mass spectrometry and is listed in Table 4.

Supplemental Figure 5C. Identification of proteins from peptides in spots 4 and 5 (see Figure 6).
UniProt Accession Q84YV8 (LxS8 Nectarin-3)

```
                   10         20         30         40         50
           MRMAAITKML FISFLFLSSV FLARSGEVDD ESEFSYDEKS ENGPANWGNI
Peptide 4c                                       YDEKS ENGPANWG
Peptide 4d                                           S ENGPANWGNI
Peptide 4e                                               GPANWGNI

                   60         70         80         90        100
           RPDWKECSGK LQSPIDIFDL RAEVVSNLRI LQKDYKPSNA TLLNRGHDIM
Peptide 4d RP
Peptide 4e RPDWK
Peptide 4f RPDWKECSGK LQS

                  110        120        130        140        150
           LRLDDGGYLK INETQYQLKQ LHWHTPSEHT INGERFNLEA HLVHESNNGK
Peptide 4h               TQYQLKQ LHWHTP
Peptide 4g                            PSEHT INGERFNL
Peptide 4a                                              LVHESNNGK
Peptide 4b                                             HLVHESNNGK
Peptide 5a                                              LVHESNNGK
Peptide 5b                                             HLVHESNNGK

                  160        170        180        190        200
           FVVIGIVYEI GLWPDPFLSM IENDLKVPAN KKGIERGIGI IDPNQIKLDG
Peptide 4a FVVI
Peptide 4b FVV
Peptide 4k               PDPFLSM IENDLK

Peptide 5a FVVI
Peptide 5b FVV

                  210        220        230        240        250
           KKYFRYIGSL TTPPCTEGVV WIIDRKVKTV TRRQIKLLQE AVHDGFETNA
Peptide 4i         SL TTPPCTEGVV W
Peptide 4n                                    RQIKLLQE AVHDG
Peptide 4j                                               HDGFETNA
Peptide 4l                                                    TNA
Peptide 5c         SL TTPPCTEGVV W

                  260        270
           RPTQPENERY INSTYHSFGI EKQQ
Peptide 4j RPTQP
Peptide 4l RPTQPENERY
Peptide 4m RPTQPENERY INS
```

Peptides 4a – 4n and 5a – 5c were identified via mass spectrometry and are listed in Table 4.
